# L-Arginine-Dependent Nitric Oxide Production in the Blood of Patients with Type 2 Diabetes: A Pilot, Five-Year Prospective Study

**DOI:** 10.3390/life14050556

**Published:** 2024-04-26

**Authors:** Irina Stoian, Liviu Iosif, Marilena Gilca, Adelina Vlad, Ioan Tivig, Ovidiu Marius Bradescu, Octavian Savu

**Affiliations:** 1 Department of Functional Sciences I/Biochemistry, Faculty of Medicine, Carol Davila University of Medicine and Pharmacy, 050474 Bucharest, Romania; irina.stoian@umfcd.ro (I.S.); liviu.iosif@umfcd.ro (L.I.); marilena.gilca@umfcd.ro (M.G.); 2 IristLabmed SRL, 031235 Bucharest, Romania; ioan.tivig@umfcd.ro; 3 Department of Functional Sciences I/Physiology, Faculty of Medicine, Carol Davila University of Medicine and Pharmacy, 050474 Bucharest, Romania; 4 Biophysics and Cellular Biotechnology Department, Excellence Center for Research in Biophysics and Cellular Biotechnology, Faculty of Medicine, Carol Davila University of Medicine and Pharmacy, 050474 Bucharest, Romania; 5 N.C. Paulescu National Institute of Diabetes, Nutrition and Metabolic Diseases, 020475 Bucharest, Romania; cercetare@paulescu.ro (O.M.B.); octavian.savu@umfcd.ro (O.S.); 6 Department of Doctoral School, Carol Davila University of Medicine and Pharmacy, 050474 Bucharest, Romania

**Keywords:** type 2 diabetes mellitus, L-arginine, arginase activity, nitric oxide, red blood cells

## Abstract

*Backgound*: Type 2 diabetes mellitus (T2DM) is a major cardiovascular risk factor. Nitric oxide (NO) is one of the many molecules that regulate vascular tone, and red blood cells (RBCs) are known to play an important role in adjusting cardiac function through NO export from RBCs. Our study prospectively investigated the L-arginine (L-arg)–nitric oxide (NO) metabolic pathway in the erythrocytes and plasma of subjects with T2DM. *Methods:* RBCs and plasma were collected from patients with T2DM (n = 10), at first clinical onset (baseline) and after five years of disease evolution (follow-up). L-arg content was assayed by competitive enzyme-linked immunoassay. Arginase activity and nitrate/nitrite levels were measured using spectrophotometry. *Results*: When compared to baseline, L-arg content decreased in RBCs and remained similar in the plasma; NO production decreased in RBCs and the plasma; and arginase activity was lower in RBCs and increased in plasma. *Conclusions*: The L-arg/NO metabolic pathway decreases in the RBCs of patients with T2DM five years after the first clinical onset. The persistent decrease in RBCs’ arginase activity fails to compensate for the sustained decrease in RBCs’ NO production in the diabetic environment. This pilot study indicates that the NO-RBC pool is depleted during the progression of the disease in the same cohort of T2DM patients.

## 1. Introduction

Type 2 diabetes mellitus (T2DM) poses a significant global health challenge, with estimates indicating that by 2050, more than 1.31 billion people may be affected by this illness [1]. Inadequately controlled, diabetes can lead to severe complications with the majority involving vascular pathologies. Therefore, there is a pressing need to uncover the fundamental disease mechanisms responsible for vascular complications, as this is essential for the development of innovative therapies specifically tailored to address these challenges [2].

Vascular impairments are predominantly linked to endothelial dysfunction, which results in diminished blood flow and long-term adverse effects on various organs, contributing to increased morbidity and mortality in individuals with diabetes [3]. The mechanisms leading to endothelial dysfunction in T2DM are complex that may explain why glucose lowering therapy has not conclusively reduced mortality in high-risk cardiovascular patients [4]. Among the various influencing factors, abnormalities in NO bioavailability and its metabolic pathway stand out as particularly important in altering endothelial homeostasis. Nitric oxide is produced within blood vessels through the activity of endothelial nitric oxide synthase (eNOS), playing a crucial role in maintaining normal vascular function and modulating vascular tone [3].

L-arginine (L-arg) is a vital precursor for the generation of nitric oxide (NO) in various human cells [5]. The origins of L-arg present in adult plasma during the post-prandial state are both exogenous (derived from the diet) and endogenous (resulting from protein degradation and citrulline) [5]. The liver, lacking an essential role in the net synthesis of L-arg, does not primarily extract either citrulline or L-arg from the bloodstream. Moreover, it exhibits exceptionally high arginase activity, a key enzyme involved in the hydrolysis of arginine into urea plus ornithine [5]. Nitric oxide can be enzymatically produced in a reaction catalyzed by three isoforms of NOS [6]. Alternatively, the non-enzymatic route of nitrite-derived NO production tends to predominate under ischemic conditions in various tissues [7], including RBCs [8].

RBCs enzymatically produce NO and citrulline from L-arg degradation, facilitated by NOS [9]. Earlier research has demonstrated that endothelial NOS is expressed in RBCs, suggesting its potential utilization as a vascular source of NO [9]. The arginase pathway is quantitatively most significant for L-arg catabolism in humans, generating urea and ornithine through two arginase isoforms [5]. Arginases serve as key enzymes in the urea cycle and act as the main competitors for NOS activity, both utilizing L-arg as a substrate [10]. Extrahepatic type 1 arginase is also expressed in RBCs [11]. Therefore, elevated arginase activity can lead to a decrease in arginine levels, diminishing its availability to endothelial NOS and subsequently reducing NO production.

In T2DM individuals, vascular dysfunction has been linked to a low plasma concentration of L-arg [12] and diminished accessibility of L-arg to eNOS, whereas arginase activity has shown a positive correlation with HbA1c levels [13]. Arginase may also contribute to the formation of reactive oxygen species (ROS) by inducing the uncoupling of NOS, a situation in which eNOS produces superoxide instead of NO. The increased levels of ROS, in turn, can further enhance the inactivation of NO [14,15].

Several studies indicate that, in comparison with healthy individuals, L-arg concentrations in the RBCs of patients with T2DM significantly decreased in advanced age [16] or at more progressed stages of the disease [17]. These levels are maintained in incipient diabetes [18] and are higher in younger cells [16]. Interestingly, a 75 g oral glucose administration to patients with prediabetes was recently reported to correlate with heightened oxidative stress, inhibition of the NO metabolic pathway, and an elevation in eryptosis (the self-destructive death of erythrocytes) in RBCs [19]. However, conflicting data arise from clinical studies employing L-arg supplementation. In a double-blind study, the administration of L-arg treatment (3 g three times per day for one month) led to a substantial improvement in peripheral and hepatic insulin sensitivity in T2DM patients, although complete normalization was not achieved [20]. In contrast, the supplementation of L-arg (3 g three times per day for six months) to improve endothelial function in a cohort of ST-elevation myocardial infarction patients, alongside standard postinfarction therapies, did not enhance vascular stiffness measurements. Additionally, it was reported to be linked to increased postinfarction mortality [21]. On the other hand, the administration of L-citrulline, a natural arginase inhibitor, has been reported to elevate plasma NO levels and decrease arginase activity in individuals with T2DM [21].

NO export from RBCs has been demonstrated to enhance vascular relaxation under hypoxic conditions [22,23] and to exert a crucial role in improving cardiac function [24]. For instance, in an acute model of myocardial ischemia/reperfusion (I/R) in chimeric mice, erythrocyte eNOS was found to be essential in reducing the infarct size, preserving cardiac function, and maintaining optimal nitric oxide levels in RBCs [25]. T2DM can impede NO-mediated vasorelaxation induced by RBCs [26] and hinder cardiac post-ischemic recovery through an RBC arginase-dependent modulation of eNOS and ROS in mice [24].

NO bioavailability varies in the plasma of patients with diabetes [27,28,29]. Our previous investigation [18] and other cross-sectional studies [27] showed a potential increase in red blood cells’ NO availability in diabetic patients at the initial clinical onset. However, there is currently a lack of information about the L-arg/NO metabolic pathway in RBCs during the progression of the disease within the same cohort of T2DM patients. Thus, in the present prospective study, we investigated the L-Arg/NO metabolic pathway in both red blood cells and the plasma of diabetic patients, at baseline and five years after the initial clinical onset.

## 2. Materials and Methods

### 2.1. Ethical Approval

The study was approved by the ethics committee of the “N. C. Paulescu” National Institute of Diabetes, Nutrition, and Metabolic Diseases, Bucharest, Romania (reference numbers 199/2009 and 165/2014, respectively). Each participant provided their signature on an informed consent form endorsed by the Ethics Committee before participating in the study. The procedures adhered to the principles outlined in the Helsinki Declaration of 1975.

### 2.2. Subjects and Study Design

**Figure 1:**
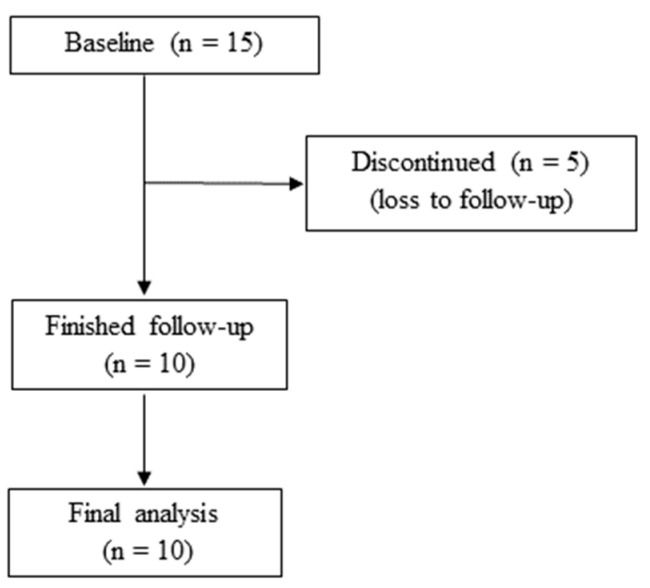
CONSORT diagram of patient flow.

In this prospective cohort study, fifteen non-insulin-dependent outpatients with T2DM attending the “N. C. Paulescu” National Institute of Diabetes, Nutrition and Metabolic Diseases in Bucharest, Romania, were enrolled between April and September 2009. Ten patients (five men and five women) remained in the study and were comparatively evaluated at the initial clinical onset (baseline) and after five years of disease progression (follow-up, September to December 2014). Figure 1 represents a CONSORT diagram of our study.

Either at baseline or follow-up, the subjects included were asymptomatic outpatients without acute hyperglycemia crisis (venous blood glucose < 200 mg/dL), inflammatory or infectious disease (white blood cell count 4000–11,000/µL; erythrocyte sedimentation rate—ESR < 20 mm/h), active liver disease (AST and ALT venous blood values 0–41 UI/L), or current immunosuppressive therapy. None of these patients had known hematological diseases. The relevant data from complete blood counts were within normal ranges (Hb values 0.7–15.0 g/dL; RBC count 4.2–5.5 × 10^6^/µL; platelet count 150,000–450,000/µL) and are provided in Table 1.

All subjects were undergoing nutritional therapy and taking oral antidiabetic drugs (metformin and sulphonylurea, n = 5; metformin, n = 5) to maintain glycemic control, aiming for a target glycated hemoglobin (HbA1c) level of <7%. The patients undertook an examination to identify specific chronic complications of diabetes (fundoscopy, urinary albumin-to-creatinine ratio, ankle-brachial index). A comprehensive medical history and a record of concurrent medications were obtained through interviews with the patients.

### 2.3. Sample Processing

Following overnight fasting, blood was sampled from *vena mediana cubiti* and anticoagulated using lithium-heparin vacutainers (BD Vacutainer, Franklin Lakes, NJ, USA). An amount of 5 mL of venous blood was collected, homogenized in a sterile centrifugation tube, and centrifuged at 2000× *g* for 15 min, at room temperature. After centrifugation, the plasma and buffy coat were separated and kept on ice, and the remaining erythrocytes were washed three times with 0.9% NaCl and lysed (1:1) in ultrapure water [30]. The samples were aliquoted, stored at −80 °C, and thawed only once before further analysis. L-arg content, arginase activity, and nitrate and nitrite analysis were performed in plasma and RBCs from all samples simultaneously.

### 2.4. Determining L-Arginine Concentration

The concentration of L-arg, a substrate of NOS and arginase, was measured using an L-arg-ELISA (Immundiagnostik AG, Bensheim, Germany), based on a competitive enzyme-linked immunoassay method. The dynamic range of the standard curve was 12.5–300 µmol/L. Plasma samples (100 µL) were mixed with ethanol (500 µL), and the proteins were precipitated after centrifugation at 10,000× *g* for 15 min, at room temperature [31]. Proteins from RBC lysates were precipitated with a mixture of chloroform/ethanol (vol/vol, 33.33/66.66) [30]. The upper, clear aqueous layer was collected. Both supernatants were then evaporated under a nitrogen stream, and the residues were re-dissolved with 350 µL of ultrapure water [32]. L-arg detection was performed in all samples following the manufacturer’s instructions. Detection was carried out using an enzymatic color reaction measured in a photometer at 450 nm. Each sample was assessed in duplicate. L-arg concentration was determined by employing a four-parameter algorithm for the dose–response curve of absorbance units, utilizing standard dilutions with defined concentrations (intra-assay coefficient of variation, CV (%) 8.9 for 94.4 umol/L L-arg; inter-assay CV (%) 3.6 for 95.4 umol/L L-arg).

### 2.5. Arginase Activity Assay

The determination of arginase activity was performed based on urea production in the sample [33]. In brief, erythrocyte lysates or plasma samples (50 µL) were initially placed in a mixture containing 0.1 M glycine buffer (pH 9.5), 0.4 M MnCl_2_, and 1 mM 2-mercaptoethanol. The mixture was then activated by incubation for 1 h at 37 °C. Subsequently, a 0.9 M arginine solution at pH 9.5 was added, and the mixture was incubated for 10 min. The reaction was stopped by adding 0.33 mmol/L perchloric acid, and the mixture was centrifuged for 15 min at 3500 rpm. To evaluate the basal urea concentration, a blank solution was prepared by dissolving each sample in a mixture containing 0.1 M glycine buffer (pH 9.5) and 0.33 mmol/L perchloric acid, followed by centrifugation for 15 min at 3500 rpm. The urea concentration in each sample supernatant was measured spectrophotometrically in triplicate at 578 nm using an automatic analyzer (Cobas Mira Plus, Roche Diagnostics, Rotkreuz, Switzerland), by the urease method, following the manufacturer’s recommendations (Dialab, Wiener Neudorf, Austria). One unit of urease activity represents the conversion of 1 µmol of urea per minute at 37 °C.

### 2.6. Nitrate and Nitrite Analysis

While nitrate and nitrite are not direct measurements of NO, they are used as reliable markers for NO production [34]. Nitrate and nitrite were determined following the protocol described by Miranda et al. [31]. Plasma samples (100 µL) were mixed with ethanol (500 µL) and the proteins were precipitated after centrifugation (10,000× *g*, 15 min, RT) [33]. RBC proteins were precipitated with a mixture of ethanol/chloroform, as previously described [30]. Nitrate and nitrite analyses were conducted spectrophotometrically, using an assay that combined the reduction of nitrate with vanadium (III) and the measurement of nitrite in a single step [34]. Reactions were carried out at 37 °C, considering that, at temperatures below 80 °C, nitrate reduction by vanadium (III) is halted following nitrite formation. Griess reagents [N-1-Naphthylethylenediamine dihydrochloride (NEDD), and sulphanylamide (SULF)] (Promega, Madison, WI, USA) were used as trapping agents for the simultaneous detection of nitrite and nitrate. Briefly, for the standard curve, a nitrate standard solution (50 µL) was serially diluted (from 100 to 5 µM) in duplicate in a 96-well flat-bottomed, polystyrene microtiter plate (Corning, New York, NY, USA). After the loading of the plate with 50 µL samples, a saturated solution of VCl_3_ (40 mg in 5 mL HCl 1 M) was added to each well, followed immediately by the addition of SULF 2% in HCl 1 M (25 µL) and NEDD 0.1% in dH_2_O (25 µL). The samples were then incubated for 40 min at 37 °C, and the total nitrite and nitrate (NOx) content was measured spectrophotometrically at 540 nm. All samples were assayed in triplicate. The VCl_3_ solution and the Griess reagents were freshly prepared immediately before application to the plate. Sample blank values were obtained by substituting the diluting medium for Griess reagent. Nitrite was similarly measured, except that the samples and nitrite standards were only exposed to Griess reagents.

### 2.7. HbA1c Assay

Hb A1c was measured by standardized immunoturbidimetry in whole blood samples (Cobas Integra, Roche Diagnostics, Rotkreuz, Switzerland), following the manufacturer’s recommendations [35].

### 2.8. Statistical Analysis

Data analysis was performed using the GraphPad Prism software package (https://www.graphpad.com/) (GraphPad Software Inc., San Diego, California, CA, USA). Between-group differences were determined using parametric (two-tailed Student’s *t*-test) or nonparametric (two-tailed Wilcoxon) tests. A *p* < 0.05 was considered statistically significant. No data imputation was performed.

## 3. Results

### 3.1. Clinical Characteristics of Diabetic Patients at Baseline and Five-Year Follow-Up

Table 1 provides a summary of the demographic and clinical features of the study participants. There were no statistical differences between biological parameters at follow-up when compared with baseline, suggesting a relatively steady biological status. All the participants in the study did not exhibit chronic complications. Four patients adhered to angiotensin-converting enzyme inhibitor (ACEI) and statin treatments for over six months.

### 3.2. Levels of L-Arginine in Plasma and Red Blood Cells

**Figure 2:**
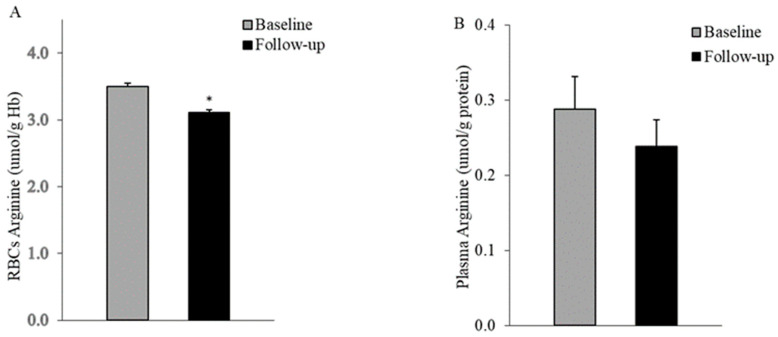
L-arginine pool is decreased in RBCs (**A**) while remaining similar in the plasma (**B**) of T2DM patients after five years of disease progression (n = 10). Values are mean ± SEM; * *p* < 0.05 between groups, paired *t*-test.

Compared to the baseline measurements, there was a notable reduction in the L-arg pool within red blood cells, as indicated by a significant decrease (3.52 ± 0.2 vs. 3.10 ± 0.2 μmol/g Hb, *p* = 0.022, paired *t*-test) shown in Figure 2A. In contrast, the L-arg levels in plasma (Figure 2B) remained stable in patients with diabetes over five years of disease progression (0.28 ± 0.04 vs. 0.24 ± 0.03 μmol/g protein, *p* = ns, respectively).

### 3.3. Arginase Activity in the Blood of Diabetic Patients

**Figure 3:**
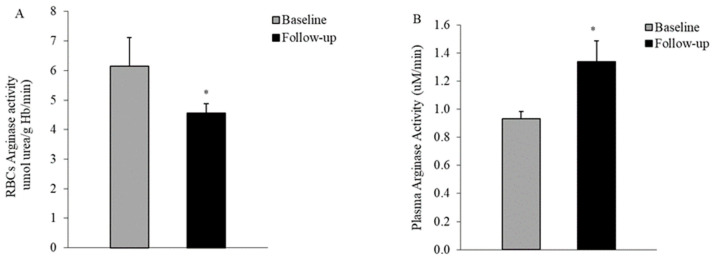
Arginase activity decreased in RBCs (**A**) and increased (**B**) in the plasma of T2DM patients (n = 10) five years after first clinical onset when compared to baseline (first clinical onset). Values are means ± SEM; * *p* < 0.05 between groups, paired *t*-test.

A consistent reduction in arginase activity was observed in the RBCs of diabetic patients at five years of disease progression compared to baseline values (6.15 ± 0.96 vs. 4.56 ± 0.5 μmol urea/g Hb/min, *p* = 0.025, paired *t*-test), as illustrated in Figure 3A. Conversely, in plasma, the enzyme’s activity increased five years after the initial clinical onset relative to baseline measurements (0.93 ± 0.05 vs. 1.33 ± 0.14 μM/min, *p* = 0.016, paired *t*-test) (Figure 3B).

### 3.4. Nitric Oxide Production in the Blood of Diabetic Patients

**Figure 4:**
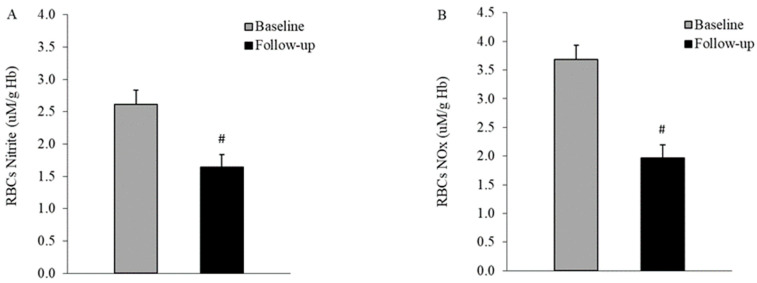
RBC NO production decreased in the RBCs of T2DM patients after five years of disease progression. Nitrite (**A**) and nitrite plus nitrate (NOx) (**B**) were measured in RBCs from subjects with T2DM at first clinical onset and after five years (n = 10). Values are expressed as means ± SEM; # *p* < 0.01 between groups, paired *t*-test.

**Figure 5:**
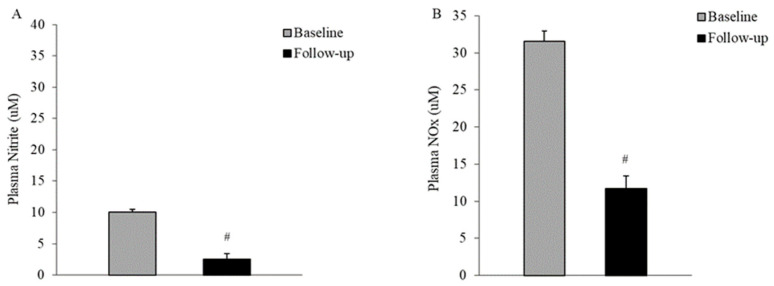
Plasma NO production decreased in the plasma of T2DM patients five years after the first clinical onset. Nitrite (**A**) and nitrite plus nitrate (NOx) (**B**) were assayed in the plasma of patients with T2DM at first clinical onset and after five years (n = 10); # *p* < 0.01 between groups, two-tailed Wilcoxon test.

NO, represented by both nitrite and total NO production, exhibited a decrease in RBCs (2.60 ± 0.23 vs. 1.64 ± 0.22 μM/g Hb, *p* = 0.007; 3.69 ± 0.36 vs. 1.97 ± 0.25 μM/g Hb, *p* = 0.001, for nitrite and nitrite plus nitrate, respectively, paired *t*-test) and plasma (10.0 ± 0.45 vs. 2.51 ± 0.15 μM, and 31.5 ± 1.38 vs. 11.6 ± 0.83 μM, for nitrite and nitrite plus nitrate, respectively, *p* < 0.01, Wilcoxon test) during the follow-up period, as depicted in Figure 4 and Figure 5, compared to the baseline.

### 3.5. Impact of Statin and ACEI Treatment on Arginase Activity and NO Synthesis

An influence of 3-hydroxy-3-methylglutaryl-coenzyme A (HMG-CoA) inhibitors and ACEI medication on arginase activity was reported in the literature [36]; therefore, we investigated the impact of such treatments on arginase activity and NO production. Analyzing the absolute differences in arginase activity and total NO production in RBCs and plasma during the five-year follow-up, we observed the evolution of these parameters among subjects treated with ACEI and statins that mirrored the changes observed in those not receiving treatment (3.46 ± 0.61 vs. 3.22 ± 1.1 μmol urea/gHb/min in red blood cells, and 0.55 ± 0.14 vs. 0.68 ± 0.23 μM/min in plasma, *p* = ns; 1.77 ± 0.32 vs. 2.25 ± 0.4 μM/gHb in RBCs and 17.7 ± 2.4 vs. 21.2 ± 2.8 μM in plasma, *p* = ns, for arginase activity and total NO, respectively). However, it should be noted that these results are underpowered due to the low number of subjects in each group (four ACEI- and statin-treated patients, and six non-treated patients).

## 4. Discussion

The current cross-sectional study presents a comparative analysis of L-arg-dependent NO production in the blood of a cohort of T2DM patients at the initial clinical onset of diabetes and five years into the progression of the disease. Our findings reveal a significant decline in the erythrocytes pool of both L-arg and NO content as the disease advances.

Several research groups have studied the impact of diabetes mellitus on L-arg concentration, yielding disparate findings. Bizjak et al. [16] conducted a cross-sectional study, reporting that the L-arg concentration in patients with T2DM was notably higher in young RBCs compared to healthy subjects but significantly reduced in older cells. On the contrary, Ramirez-Zamora et al. [17] observed lower L-arg levels in the RBCs of individuals with T2DM at various stages of the disease, in comparison with healthy individuals. However, Gajecki et al. [37], in a recent cross-sectional study, found a concentration of L-arg in the erythrocytes of patients in the early stages of T2DM similar to healthy controls, and our earlier cross-sectional data [18] suggested that the L-arg pool from RBCs is not yet depleted in patients with T2DM at the initial clinical onset. These findings collectively underscore the dynamic changes in L-arginine levels at various stages of type 2 diabetes, emphasizing the complexity of its metabolism concerning disease progression. Furthermore, they highlight the necessity of conducting longitudinal investigations within the same cohort—a gap that our study addressed for the first time.

Our current results suggest that the L-arg levels in RBCs are depleted relatively early in the course of diabetes progression (Figure 2A). Additionally, five years after the clinical onset, arginase activity was decreased in the RBCs of patients with T2DM (Figure 3A). These data are in line with our earlier cross-sectional investigation [18] that indicated a decrease in this enzyme’s activity in the RBCs of patients with T2DM at the initial clinical onset. A decreased activity of an RBC-NOS competitor, arginase I, during RBC aging, was reported as well in a cross-sectional comparison between healthy controls and subjects with T2DM. Arginase activity was lower in the oldest RBC fractions isolated from subjects with diabetes. However, another study [17] reported increased arginase affinities for arginine in the RBCs of patients with T2DM at various stages of the disease, demonstrating a net arginine catabolism by RBCs. Likewise, in a recent investigation, Zhou et al. [38] reported a significant upregulation of arginase activity and protein expression in the RBCs of T2DM patients, which has been proposed to be mediated by ROS overproduction.

Consequently, the consistent decrease in RBC arginase activity over the five years of disease progression, revealed by our longitudinal investigation in the same cohort of non-insulin-dependent diabetic patients, might indicate an adaptive response against endothelial dysfunction. This dysfunction is often linked to the observed depletion of NO in the diabetes milieu. Considering the competition with NOS for the substrate L-arg, the upregulation of arginase in endothelial cells inhibits the production of NO derived from eNOS, contributing to the characteristic endothelial damage in diabetes [39].

Assays performed in the plasma of our subjects indicated a decrease in L-arg levels (Figure 2B) and an increase in arginase activity (Figure 3B) after five years of disease progression. These findings are consistent with previous cross-sectional reports [12,37], affirming heightened arginine catabolism in the plasma of patients with T2DM. Kosenko et al. [36] reported that neither lisinopril nor simvastatin succeeded in reducing increased arginase activity in the plasma of patients with hypertension and hyperlipidemia. Indeed, in our diabetic subjects, despite the reduced sample number, we noted similar differences in activity values at baseline and follow-up between the treated and non-treated groups.

Further, our study reported a decrease in NO production in RBCs five years after the initial clinical onset of T2DM (Figure 4). Previous research has indicated an increase in nitrite concentration in RBCs from patients with T2DM during cellular aging [16], at the initial clinical onset [18], or at various stages of the disease [17]. Our data suggest that the RBC pool becomes depleted during the progression of diabetes. While less probable, the potential deleterious effect of storage on RBCs’ NO metabolism could not be excluded, specifically, a gradual decrease with the rapid stabilization of nitrite concentrations, in contrast to the steady nitrate levels in RBCs from whole blood stored for 42 days at 4 °C [40].

In the plasma, over the five-year follow-up, there was a significant decrease in NO metabolites compared to the disease onset (baseline) (Figure 5). These results align with previous research [28,37]. Patients with T2DM in the early stages of the disease presented notable differences in the nitric oxide metabolic pathway compared to healthy counterparts, especially within the plasma compartment, as reported by Gajecki et al. [37]. They hypothesized that erythrocytes act as a protective buffer with higher NO bioavailability, experiencing less of an impact from diabetes mellitus-related deviations. Despite numerous inconclusive reports [17,27,41], both the findings of Gajecki et al. [37] and our observations support the notion of erythrocytes acting as a more stable reservoir for NO in the context of diabetes mellitus, when compared to healthy individuals or longitudinally, tracking the course of disease progression within the same cohort, respectively.

It is noteworthy that a cross-talk connection between the erythrocyte L-arg/NO metabolic pathway and endothelial function in individuals diagnosed with T2DM was previously revealed [37]. The analysis of biochemical findings and endothelial function demonstrated moderate correlations between increased arterial stiffness and impaired endothelial function on one hand and NO bioavailability in the RBCs’ compartment on the other hand, while no such correlations were observed in the plasma of patients with T2DM [37].

Several limitations of the current research should be acknowledged. The number of subjects included in our study was relatively small, attributed to limited resources and patient withdrawals throughout the follow-up period. Nonetheless, our research was intended as a pilot longitudinal study, and the obtained data have reached statistical significance. Another limitation concerns the molecules under examination. Due to the facile reactivity of NO with hemoglobin, direct measurement proved challenging, and consequently, our findings rely on more stable by-products. Furthermore, it is noteworthy that our investigation focused on subjects with newly onset/early diabetes, without the concomitant cardiovascular disorders or vascular complications of T2DM, suggesting that our results should not be readily generalized to the entire diabetic population.

## 5. Conclusions

Although the sample size of this pilot, five-year prospective study is rather limited, our findings suggest a reduction in NO availability within RBCs, linked to dysregulation of arginine metabolism during the progression of the disease in the same cohort of T2DM patients. We observed contrasting activities of arginase in red blood cells compared to plasma as the disease advanced. As demonstrated in previous cross-sectional studies comparing patients with T2DM to healthy subjects [12], this discrepancy may be attributed to the presence of different arginase isoforms in these distinct blood compartments. Therefore, the persistent decline in arginase activity observed in RBCs throughout diabetes progression could represent an adaptive response that ultimately proves insufficient to compensate for the NO depletion-mediated endothelial dysfunction characteristic of the diabetic milieu.

## Figures and Tables

**Table 1:** Patients’ characteristics.

	Type 2 Diabetes Mellitus		*t*-Test (Paired)
	Baseline	Follow-Up (+5 Years)	
Age (years)	50.3 ± 3.0	56.0 ± 3.1	
Gender (n, women/men)	5/5	5/5	
BMI (kg/m^2^)	26.9 ± 1.0	27.3 ± 0.9	0.53
OAD (n)	5	5	N/A
SBP (mmHg)	140.1 ± 5.0	142.0 ± 4.1	0.81
DBP (mmHg)	87.1 ± 2.1	83.1 ± 1.7	0.13
HbA1c (%)	8.4 ± 0.6	8.6 ± 0.5	0.80
Hb (g/dL)	14.4 ± 0.30	14.5 ± 0.50	0.88
RBCs (×10^5^/µL)	4.95 ± 0.08	4.9 ± 0.12	0.83
WBCs (×10^3^/µL)	7.35 ± 0.3	7.3 ± 0.3	0.35
PLT (×10^3^/µL)	230.1 ± 14.9	221.3 ± 15.2	0.68
Cholesterol (mg/dL)	213.3 ± 12.0	212.5 ± 21.1	0.97
HDL (mg/dL)	38.5 ± 3.3	46.6 ± 2.9	0.09
TG (mg/dL)	188.6 ± 26.0	175.5 ± 19.8	0.69
Creatinine (mg/dL)	0.81 ± 0.1	0.73 ± 0.1	0.39
ALAT (UI/L)	28.2 ± 6.7	24.7 ± 3.1	0.59
ASAT (UI/L)	51.6 ± 11.7	42.4 ± 5.2	0.39

Values are mean SEM. SBP—systolic blood pressure; DBP—diastolic blood pressure; HbA1c—hemoglobin A1c; Hb—hemoglobin; RBCs—red blood cells; WBCs—white blood cells; PLT—platelet; TG—triglycerides; HDL—high-density lipoproteins; ALAT—Alanine Aminotransferase; ASAT—Aspartate Aminotransferase; OAD—oral antidiabetics; BMI—Body Mass Index.

## Data Availability

Data available on request.
